# Persistent sex disparities in clinical outcomes with percutaneous coronary intervention: Insights from 6.6 million PCI procedures in the United States

**DOI:** 10.1371/journal.pone.0203325

**Published:** 2018-09-04

**Authors:** Jessica Potts, Alex Sirker, Sara C. Martinez, Martha Gulati, Mirvat Alasnag, Muhammad Rashid, Chun Shing Kwok, Joie Ensor, Danielle L. Burke, Richard D. Riley, Lene Holmvang, Mamas A. Mamas

**Affiliations:** 1 Keele Cardiovascular Research Group, Centre for Prognosis Research, Institute of Primary Care and Health Sciences, Keele University, Stoke-on-Trent, United Kingdom; 2 University College London Hospitals and St Bartholomew's Hospital, London, United Kingdom; 3 Division of Cardiology, Providence St. Peter Hospital, Olympia, Washington, United States of America; 4 Division of Cardiology, University of Arizona, Phoenix, AZ, United States of America; 5 King Fahd Armed Forces Hospital, Jeddah, Saudi Arabia; 6 Department of Cardiology, Royal Stoke Hospital, Stoke-on-Trent, United Kingdom; 7 Department of Cardiology, Rigshospitalet, Copenhagen University Hospital, Copenhagen, Denmark; University of Tampere, FINLAND

## Abstract

**Background:**

Prior studies have reported inconsistencies in the baseline risk profile, comorbidity burden and their association with clinical outcomes in women compared to men. More importantly, there is limited data around the sex differences and how these have changed over time in contemporary percutaneous coronary intervention (PCI) practice.

**Methods and results:**

We used the Nationwide Inpatient Sample to identify all PCI procedures based on ICD-9 procedure codes in the United States between 2004–2014 in adult patients. Descriptive statistics were used to describe sex-based differences in baseline characteristics and comorbidity burden of patients. Multivariable logistic regressions were used to investigate the association between these differences and in-hospital mortality, complications, length of stay and total hospital charges. Among 6,601,526 patients, 66% were men and 33% were women. Women were more likely to be admitted with diagnosis of NSTEMI (non-ST elevation acute myocardial infarction), were on average 5 years older (median age 68 compared to 63) and had higher burden of comorbidity defined by Charlson score ≥3. Women also had higher in-hospital crude mortality (2.0% vs 1.4%) and any complications compared to men (11.1% vs 7.0%). These trends persisted in our adjusted analyses where women had a significant increase in the odds of in-hospital mortality men (OR 1.20 (95% CI 1.16,1.23) and major bleeding (OR 1.81 (95% CI 1.77,1.86).

**Conclusion:**

In this national unselected contemporary PCI cohort, there are significant sex-based differences in presentation, baseline characteristics and comorbidity burden. These differences do not fully account for the higher in-hospital mortality and procedural complications observed in women.

## Introduction

Disparities in clinical outcomes between men and women undergoing percutaneous coronary intervention (PCI) have been reported in the literature [1–4]. Several studies have found a greater incidence of adverse clinical events (including higher in-hospital mortality) in women following PCI [5, 6]. More recent pooled evidence, derived from large clinical trials of modern drug eluting stent (DES) platforms, indicates contemporary DES are associated with similar safety and efficacy profiles between men and women, and factors beyond the stents themselves are likely to be more relevant [7]. Much of the higher unadjusted risk described for PCI in women can be attributed to consistent confounders in study populations–particularly older age and greater comorbidity, compared to male counterparts [8]. However, findings from separate adjusted studies evaluating sex disparity in PCI are inconsistent [4, 6, 9–14]. For example, a recent nationwide observational report from Germany, involving over 185,000 patients, identified a higher adjusted in-hospital mortality risk in women who present with ST elevation myocardial infarction (STEMI) [15]. In contrast, the observational TRANSLATE-ACS study in 12,000 ACS patients undergoing PCI from the United States found that significantly higher unadjusted 1 year major adverse cardiac events (MACE) seen in women presenting with acute myocardial infarction disappeared after adjustment for confounders [16]. Similarly, in the FAST-MI French registry, In-hospital mortality did not differ according to sex, irrespective of age group and at 5 years, overall and post-discharge mortality were similar in men and women [17]. A recent study undertaken in ACS patients suggests that sex disparities are mainly observed in younger patients, although they disappear as patients age [12].

The National Inpatient Sample offers an opportunity to re-evaluate this important question in the setting of a large, contemporary cohort of over 6 million U.S. patients undergoing PCI in a ‘real-world’ setting of PCI practice nationally. In this review, we examine temporal trends in clinical characteristics, indications for PCI and clinical outcomes stratified by sex over a 10-year period, and study whether the prognostic association of sex with clinical outcomes following PCI has changed over time in this large national dataset.

## Methods and results

### Data source

The data for this project was obtained from the National Inpatient Sample (NIS) for hospital discharges in the United States between 2004 and 2014. The NIS is the largest all-payer inpatient health care database, developed by Healthcare Cost and Utilization Project (HCUP), which is sponsored by the Agency for Healthcare Research and Quality (AHRQ). This project used a subset of data from the NIS database, which includes information obtained from 7 to 8 million hospital discharges per year. Before 2012, the NIS retained all discharges from a sample of hospitals; however, the sampling strategy has changed over time. Now, the NIS samples discharges from all hospitals participating in HUCP, approximating a 20% stratified sample of all discharges from US community hospitals. This change in sampling strategy aimed to reduce sampling bias and produce more generalizable results.

### Study design

We identified all individuals who had undergone a PCI between January 2004 and December 2014 by identifying all eligible discharges with an International Classification of Diseases, Ninth Revision, Clinical Modification (ICD-9-CM) procedure code of 00.66 (Percutaneous Transluminal Coronary Angioplasty), 36.06 (Insertion of non-drug-eluting coronary artery stent(s)), or 36.07 (Insertion of a drug-eluting coronary artery stent(s)). Before a revision of the codes in 2005, the codes 36.01 (Single vessel percutaneous transluminal coronary angioplasty or coronary atherectomy without mention of thrombolytic agent), 36.02 (Single vessel percutaneous transluminal coronary angioplasty or coronary atherectomy with mention of thrombolytic agent) and 36.05 (Multiple vessel percutaneous transluminal coronary angioplasty [PTCA] or coronary atherectomy performed during the same operation, with or without mention of thrombolytic agent) were also used and so these codes were also included when identifying procedures in discharges from 2004 and 2005.

All records were eligible for inclusion providing the discharge record showed that the patient had undergone a PCI procedure during their hospital stay and was over the age of 18. Patient demographics were recorded for each hospital discharge including data regarding age, patient sex, ethnicity, admission type (elective or emergent), admission day (weekday or weekend), median household income according to ZIP code, and patient comorbidity conditions using Deyo modification of Charlson comorbidity Index (CCI) [18]. The CCI was derived using a point based system with each weighted value depending on the prognostic impact of the comorbidity, with scores ranging from 1 to 6. These scores are summated to calculate the overall CCI values, which were then categorised as 0, 1, 2 or 3 or more to represent no, mild, moderate and severe comorbid burden respectively.

Each discharge record had information on up to 30 diagnoses that the patient had been given (15 between 2004 and 2008, 25 between 2009 and 2013 and 30 in 2014), and it was these diagnosis codes that were used to identify each of the comorbidity conditions present in the record in order to calculate the CCI for comorbidity burden during hospitalisation [19]. Details of ICD 9-CM codes and the included comorbidities the score used can be seen in S1 Table. These codes were also used to identify whether the patient had a primary diagnosis of an acute myocardial infarction, also if the patient had received a diagnosis of ST-elevation myocardial infarction (STEMI), non-STEMI or unstable angina at any point in their hospitalisation. They were also used to assess whether the individual had been diagnosed with cardiogenic shock.

Finally, information about the PCI procedure was determined from the procedure codes, detailing whether the PCI was single-vessel or multi-vessel, including bifurcation lesions. The use of adjunctive devices including intracoronary pressure wire, intravascular ultrasound and an assist device (such as an intra-aortic balloon pump) were also recorded (S2 Table). Where available from the procedure codes we also included the stent type deployed (bare metal, drug-eluting).

### Outcomes

In-hospital clinical outcomes and complications were identified. The main outcomes chosen included: (a) in-hospital mortality, (b) a vascular complication, (c) a cardiac complication, (d) a stroke or cerebrovascular event, (e) a bleeding complication or (f) a composite of any of the considered complications. We were also interested in the length of stay of the individuals and the total charge of hospitalisation for each record, however no statistical modelling was conducted on these. The total charge given in the dataset represents the amount that the hospital billed for the services, but it is not representative of the true cost of hospital services. Therefore, a charge-to-cost conversion ratio was used to convert the reported charges into the actual cost for the payer.

Complications were identified using ICD-9-CM codes and patient safety indicators, including: post-operative haemorrhage requiring transfusion, vascular injuries, cardiac complications including iatrogenic and pericardial complications, whether an individual required bailout or emergency coronary artery bypass grafting, post-operative stroke or transient ischaemic attack. Finally, bleeding complications were identified, including gastrointestinal, retroperitoneal, intracranial, intracerebral haemorrhage, unspecified haemorrhage, and whether a blood transfusion was required. Complications were identified by ICD-9-CM codes in any secondary diagnosis field (DX2-DX30) or through any procedural code on the record (S3 Table) [20].

### Statistical analysis

Statistical analysis was performed on Stata 14.0 (College Station, TX). Descriptive statistics are provided by each of the years included from the NIS database. Continuous variables are presented as median and interquartile range due to skewed data. Categorical data are presented as number and percentage. Differences were tested using a chi^2^ test for categorical variable and a Kruskal Wallis test for continuous variables. Where missing data was less than 10% of the covariate data, the observations with missing data were removed. Data was assumed to be missing at random. For data where more than 10% of data was missing, these covariates were not considered for inclusion in the analysis.

For all analyses, a weighting was applied to each observation (by using the svy prefix in analyses conducted in Stata). This decision followed the recommendations from AHRQ for analysis of survey data to account for the complex survey design of the NIS database. As records were not sampled individually but by hospital number, clustering of records within hospitals was taken into account in the survey estimation. This was done by defining each hospital to be the primary sampling unit. For calculation of national estimates and correct variances, sampling weights for each individual discharge that were provided by the AHRQ were used. The use of sampling weights are required because the design of the study means that different observations may have different probabilities of selection. Due to the redesign of the NIS data and the alternative sampling strategy used before 2012, these weights needed to be updated from the original sampling weights for 2004–2011 in order for the analysis to be conducted across all included years, this was done using new weights provided by AHRQ.

A multivariable analysis was conducted to examine the prognostic association (effect) of sex with (a) in-hospital mortality or (b) a composite of any defined complication and (c) each individual complication, after adjustment for all potential confounders that were measured. These were age, median income, elective admission, day of admission (weekend/weekday), primary diagnosis of MI, diagnosis of STEMI/ NSTEMI or unstable angina, diagnosis of shock, hypertension, or hypercholesterolemia, patient smoking status, Charlson comorbidities, previous PCI, previous CABG, use of an assist device or IABP, use of a bare metal or drug eluting stent, bifurcation stenting, fractional flow reserve, single or multi-vessel PCI and year of hospitalisation. Logistic regression models were fitted using maximum likelihood estimation to investigate the association of sex with in-hospital death or an in-hospital complication, either post-operative bleeding, vascular complication, cardiac complication or a stroke/TIA.

As well as considering the effect of patient sex on mortality across all years of the study, each year had the same multivariable model fitted individually, to assess whether the increased risk in women is consistent across all years of the study and whether there is a trend in the odds of mortality. Sensitivity analyses were conducted in order to understand how patients who were admitted electively, or presented with ACS differ in the effect on in-hospital mortality and complications. All analyses were conducted in Stata 14, with statistical significance measured at a 5% level, p<0.05.

#### Ethical approval and informed consent

The study is an analysis of anonymized data and ethical approval and informed consent was not required.

## Results

### Clinical characteristics at baseline

A total of 6,601,526 episodes between 2004–2014 were recorded with a procedure code indicating that a PCI had been performed during hospitalisation. Records with missing data for included outcomes were removed, death (0.02%), and hospitalisation costs (4.9%) as well as covariates of age (0.003%), sex (0.008%), elective surgery indication (0.3%) (S1 Fig). There was 7.3% of the data removed to missing data in the covariates. Ethnicity had nearly 20% missing data, and therefore missing data were not deleted but the covariate was not used in the statistical analysis. There were 4,379,093 PCIs undertaken in men (approximately 66% of the records) and 2,222,433 PCIs undertaken in women. Table 1 details the patient demographics stratified by men and women. It can be seen that women, are on average 5 years older than men. The two groups are comparable on several demographics including: ethnicity, admission day and type, single or multiple vessel PCI, and stent type used. There were similar numbers of patients with a primary diagnosis of acute myocardial infarction in both groups but a higher percentage of men were diagnosed with STEMI than women. Conversely, there was a higher percentage of women than men diagnosed with NSTEMI.

**Table 1:** Patient demographics and procedural characteristics of included records stratified by sex.

	Men	Women	P value
PCI discharges	4 379 093 (66.3%)	2 222 433 (33.7%)	
Median age, years [Interquartile Range (IQR)]	63 [54,72]	68 [59,77]	<0.001
Ethnicity			<0.001
White	64.7%	61.9%	
Black	5.4%	8.6%	
Hispanic	5.4%	5.5%	
Asian/Pacific Islander	1.8%	1.5%	
Native American	0.4%	0.4%	
Other	3.0%	2.5%	
Missing Information	19.3%	19.5%	
Elective admission	27.2%	26.9%	<0.001
Admission Day, Weekday	83.8%	84.1%	<0.001
Length of stay, median [IQR]	2 [1,3]	2 [1,4]	<0.001
Total charge, median [IQR]	$17,415 [$12,978,$24,125]	$17,537 [$12,956,$24,646]	<0.001
Median ZIP income			<0.001
1st quartile	24.8%	29.2%	
2nd quartile	26.3%	27.8%	
3rd quartile	25.2%	24.1%	
4th quartile	23.7%	18.9%	
Single vessel PCI	72.6%	73.5%	<0.001
Multi-vessel PCI	18.3%	17.0%	<0.001
Unknown vessel number	9.1%	9.5%	<0.001
Bifurcation stenting	1.8%	1.6%	<0.001
Use of assist devise or IABP	3.4%	3.0%	<0.001
Shock	2.7%	2.9%	<0.001
Primary diagnosis AMI	42.2%	38.9%	<0.001
STEMI	22.6%	18.5%	<0.001
NSTEMI	22.2%	23.8%	<0.001
Unstable angina	21.5%	23.4%	<0.001
Fractional flow reserve	0.7%	0.7%	0.89
Intravascular ultrasound	4.8%	5.1%	<0.001
Bare Metal Stent	22.4%	21.4%	<0.001
Drug Eluting Stent	73.1%	73.3%	<0.001
Unknown Stent Type	6.8%	7.3%	<0.001
Both stent types used	2.3%	2.0%	<0.001
CCI Score			<0.001
0	44.3%	35.7%	
1	33.6%	35.7%	
2	14.4%	18.3%	
≥3	7.7%	10.3%	
Hypertension	67.8%	73.6%	<0.001
Hypercholesterolemia	13.6%	13.2%	<0.001
Smoking—Yes	38.5%	29.4%	<0.001
Previous PCI	19.7%	17.2%	
Previous CABG	8.1%	6.0%	
CCI Components			
Previous MI	14.2%	11.3%	<0.001
Heart failure	13.9%	19.0%	<0.001
Peripheral vascular disease	1.4%	1.1%	<0.001
Previous stroke	3.4%	4.7%	<0.001
Dementia	0.1%	0.2%	<0.001
Chronic obstructive disease	13.8%	19.1%	<0.001
Connective tissue disease	0.9%	3.0%	<0.001
Peptic ulcer	0.6%	0.9%	<0.001
Mild liver disease	0.3%	0.3%	0.043
Moderate-severe liver disease	0.1%	0.1%	0.065
Hemiplegia	0.2%	0.2%	<0.001
Moderate-severe kidney disease	0.5%	0.7%	<0.001
Diabetes–controlled	27.6%	33.1%	<0.001
Diabetes–uncontrolled	2.8%	4.3%	<0.001
Leukaemia & lymphoma	2.0%	1.6%	<0.001
Solid tumour + metastasis	0.3%	0.3%	0.556
AIDS	0.1%	0.0%	<0.001

Significant sex differences in comorbidity burden, defined by the categorised Charlson Comorbidity Index (CCI), were evident. Men had a greater prevalence of either no or low comorbid burden (as defined by CCI scores of 0 and 1), whilst women had a greater prevalence of moderate and severe comorbid burden (as defined by CCI score of 2 and ≥3). There were a greater number of women with conditions such as heart failure, chronic obstructive disease and previous stroke, whereas more men had a history of previous MI when they were admitted.

**Fig 1:**
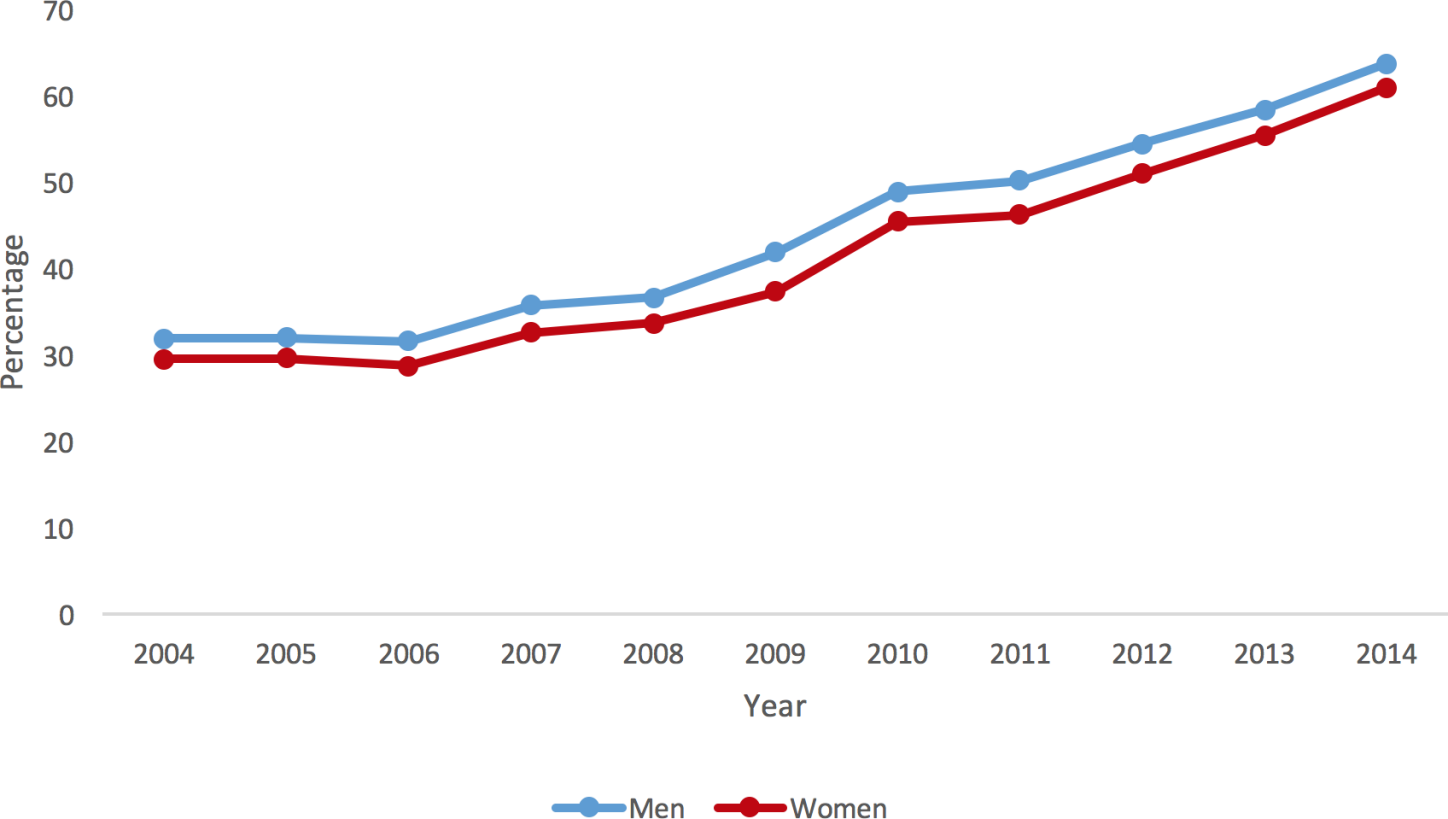
Percentage of patients each year undergoing a PCI admitted with a primary MI diagnosis, stratified by sex.

Patient demographics for each year (2004–2014) stratified by men and women for patients undergoing PCI are presented in S4 Table. Women are consistently older than men across the included years. The proportion of cases undertaken for elective indications has declined over time, across both sexes. The number of patients with a primary diagnosis of MI has increased over time across both men and women, as can be seen in Fig 1. Cardiovascular risk factors such as diabetes, heart failure, hypertension and smoking has increased over time in both men and women. With the exception of smoking, all other risk factors maintain a greater prevalence in women across all years studied. The burden of comorbidity as defined by the CCI has increased over time, although women have consistently greater comorbid burden for each year studied, as illustrated in S2 Fig.

### Clinical outcomes

**Fig 2:**
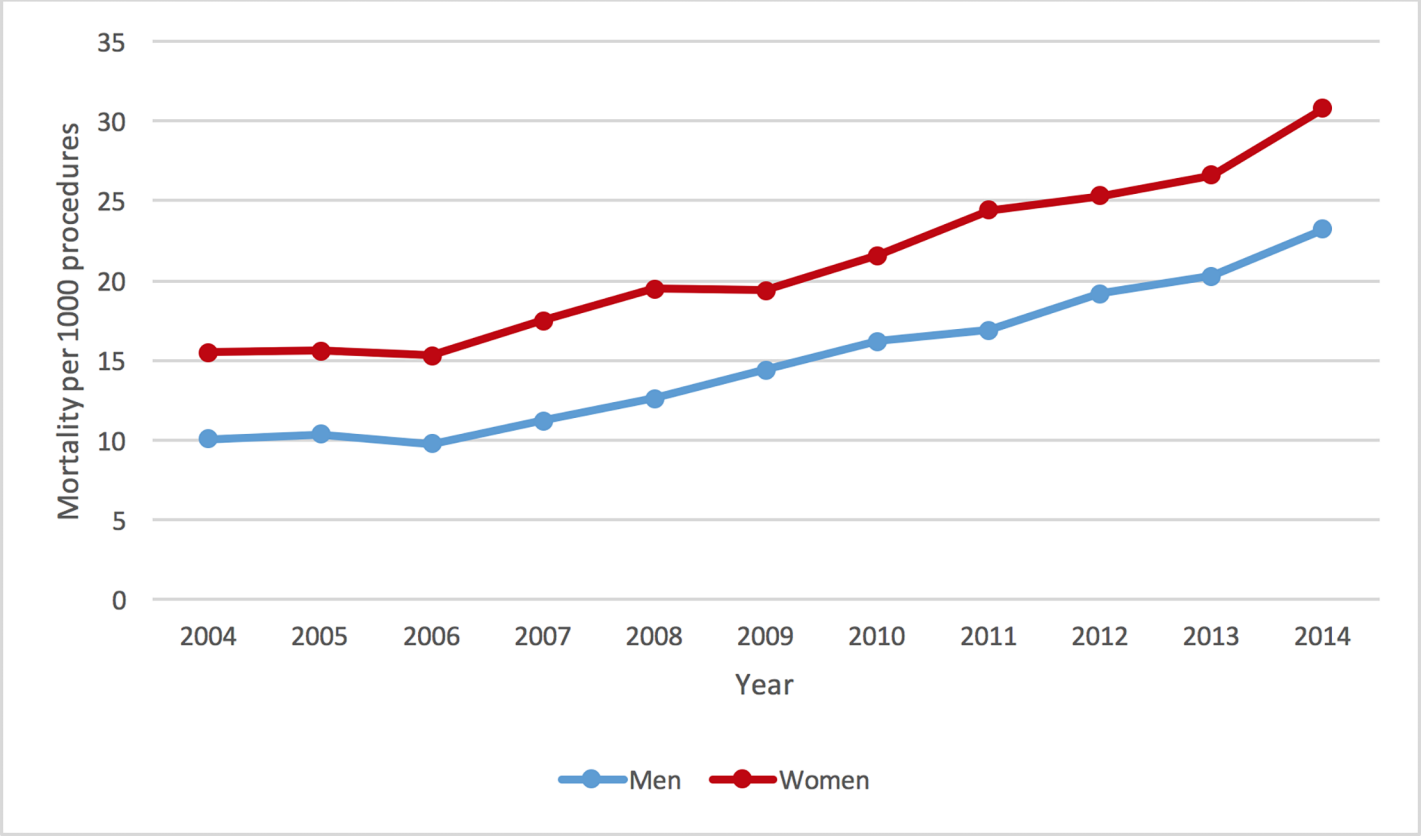
Annual rates of mortality for men and women per 1000 records.

Table 2 shows the percentages of records for which there is an in-hospital death or complication recorded, including vascular and cardiac complications and post-operative stroke/TIA or bleeding episode. Women have higher total event rates, with the exception that men have higher event rates for a post-operative stroke or a pericardial complication. Fig 2 shows the post-PCI percentage of in-hospital mortality for men and women annually (defined by year of admission) in the included study years.

**Table 2:** In-hospitality and post-procedural complications stratified by sex.

	Men	Women
**Number of PCI procedures**	4 379 093	2 222 433
**Death**	1.4%	2.0%
**Any complication**	7.8%	11.6%
**Bleeding Complications**	2.4%	5.1%
**Vascular complications**	0.9%	1.4%
Post-op haemorrhage requiring transfusion	0.01%	0.01%
Vascular injury	0.8%	1.4%
**Cardiac complications**	3.0%	3.0%
Iatrogenic cardiac	1.9%	2.1%
Pericardial comp	0.06%	0.01%
Requiring CABG	1.3%	1.0%
**Post-operative stroke/TIA** 𝛉	1.7%	1.5%

ⁱ Transient Ischaemic Attack

**Fig 3:**
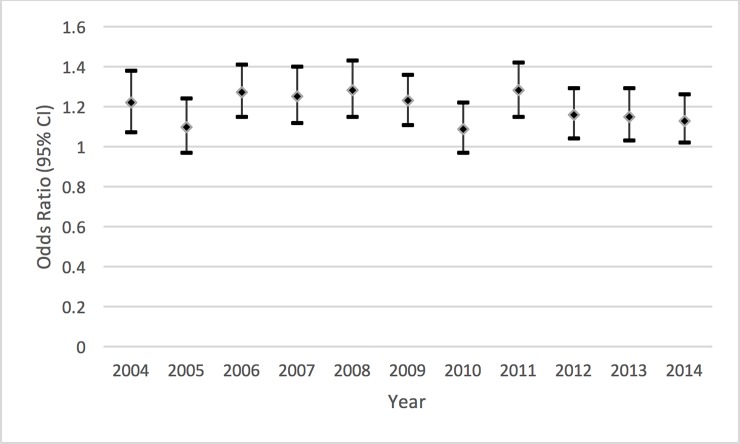
Adjusted annual odds ratio for in-hospital mortality for women versus men, defined by year of admission.

Multivariable analyses were conducted to look at the adjusted prognostic association of patient sex on in-hospital mortality and post-procedural complications with 6,601,526 discharge records included in the analysis (Table 3). Adjusted for demographic and procedural parameters, women had a significant increase in the odds of in hospital mortality and all complications. Women had a just over a 20% increase in mortality compared to men (OR 1.20 (95% CI 1.16, 1.23) after adjustment. The largest difference was seen in the bleeding complication where women had an 80% increase in the odds of a bleeding complication compared to men (OR 1.81 (95% CI 1.77, 1.86). There was a 53% increase in the odds of a vascular complication for women compared to men (1.53 (95% CI 1.47, 1.59). Fig 3 shows the annual adjusted odds ratios and 95% confidence intervals for mortality in women compared to men, with consistently greater odds of mortality amongst women across all year considered. The annual odds ratios show a statistically significant difference across all years, with the exception of 2005 & 2010, which are the only years where the odds calculated was not statistically significant.

**Table 3:** Adjusted odds ratios* and 95% confidence intervals for in-hospital mortality and complications for women versus men.

	Odds Ratio (95% CI)
In hospital mortality	1.20 (1.16,1.23)
Any complication	1.36 (1.34,1.39)
Bleeding complication	1.81 (1.77,1.86)
Vascular complication	1.53 (1.47,1.59)
Cardiac complication	0.99 (0.96,1.01)
Post-operative stroke	1.23 (1.20,1.26)

*Adjustment for age, median income, elective admission, day of admission (weekend/weekday), primary diagnosis of MI, diagnosis of STEMI/ NSTEMI or unstable angina, diagnosis of shock, hypertension, or hypercholesterolemia, patient smoking status, Charlson comorbidities, previous PCI, previous CABG, use of an assist device or IABP, use of a bare metal or drug eluting stent, bifurcation stenting, fractional flow reserve, single or multi-vessel PCI and year of hospitalisation

S5 Table gives the odds ratios and associated confidence intervals for each of the subgroups that were considered: elective procedures or a diagnosis of ACS. Women who were admitted for an elective procedure had greater odds compared to men of mortality, bleeding, vascular and cardiac complications, whereas the odds were larger for a composite of any complication and a post-operative stroke for women compared to men in the ACS population.

## Discussion

Our study shows that in a national unselected contemporary PCI cohort, there are significant sex-based differences in presentation, baseline characteristics and comorbidity burden as well as crude outcomes. These differences in clinical outcomes, persist even after adjustment for potential confounders and show that women are more likely to die in-hospital or suffer a complication than men. This difference is seen across all of the decade studied, and across both elective and ACS indications for PCI.

The questions of whether and how the patient’s sex might directly influence outcomes after PCI remain controversial. Prior, multiple, studies have yielded widely divergent results, despite the use of varied methods to mitigate confounding clinical factors [1]. This unique study reports temporal trends and significant differences in gender outcomes of more than 6.6 million patients who have undergone PCI in a decade of data from the National Inpatient Sample (NIS) within the US. Around one third of the patients who underwent PCI were women, comprising in excess of 2.2 million hospital discharges and represents the largest study to date. In addition to patient volumes, the strength of the NIS also stems from the diverse geographic and population composition, through the sampling strategy from community hospitals across the United States, reflecting real-world clinical practice. Furthermore, the inclusion of a broad range of comorbidity data contained within the NIS facilitates attempts to identify possible confounding influences on noted outcomes.

Consistent with many previous studies in this area, women undergoing PCI were significantly older than men and had a greater prevalence of comorbidity. Our observation that women were on average 5 years older than men may relate to delayed onset of symptomatic severe coronary disease in women but may also reflect delays in the diagnoses of CAD in women [21]. Women had greater cardiovascular and non-cardiovascular comorbid burden than men, across a wide variety of comorbid conditions. The greater comorbidity burden in women may relate (in part) to higher mean age of women undergoing PCI, and potentially reflect known differences between men and women in the threshold for diagnosis of coronary disease and for referral for angiography and revascularization [22].

The second noteworthy result from our work is the steadily increasing burden of comorbidity over time, and with that trend an increase in procedural mortality. In fact, it is seen that this applied similarly to both sexes and is likely to reflect a general US population that is both increasing in (mean) age and therefore accompanying comorbidity. The increased use of PCI in older patients over time will be relevant, as prior studies have revealed increased mortality among the elderly and frail with accompanying comorbidity undergoing PCI [23–25]. This combination of factors probably underlies the increase in annual in-hospital mortality seen over the study period. Another important contribution to the mortality rise is the increasing proportion of cases of acute myocardial infarction, with much higher associated risks for (in-hospital and later) MACE and mortality compared to stable ischemic heart disease presentations.

Our central finding was a higher incidence of in-hospital mortality and in-hospital complications (including bleeding and vascular problems) in women compared to men, after adjustment for available confounding demographic, comorbidity, procedural factors. Furthermore, we note that differences in in-hospital mortality have persisted over time with women consistently at 20% greater risk compared to men, even once differences in clinical characteristics and comorbid burden have been adjusted for. The present study is, to our knowledge, the largest contemporary work in this area. A number of possible explanations for this sex disparity exist. Firstly, a differing likelihood of correct diagnosis and appropriate referral for PCI between sexes has been mentioned, and is likely to be particularly important in acute presentations such as myocardial infarction, with time-sensitive outcomes from evidence-based treatments [26]. Secondly, a greater risk for bleeding appears, to some extent, inherent in female patients, as reflected in its use on bleeding risk scores [27]. This may relate to sex differences in responsiveness to antiplatelet and other therapies [28]. Increase in radial access mitigates bleeding risks in women as for men [29, 30] but higher levels of cross-over to the femoral approach in women has been noted in studies, which may therefore contribute to higher periprocedural risk [31].

A final point worthy of discussion is the change in relative risk between sexes over the time period of the study. It is striking that, whilst there has been a steady increase over time of in-hospital mortality after PCI, the risk ratio for women compared to men has remained fairly stable. This probably reflects the persistence of adverse factors noted above, which disadvantage female patients in the setting of PCI, superimposed upon the profile of steadily increasing age and comorbidity that is affecting both sexes. As such, this pattern underscores the urgent need for more focussed efforts to address these residual sex-specific issues, which maintain sex disparities distinct from the changing background of overall outcomes.

A number of limitations in our study exist, which arise due to the use of existing, routinely collected data. The outcome measures available from the National Inpatient Sample relate only to in-hospital outcomes. A more complete picture would be provided by longer-term follow-up of mortality and other adverse events. Whilst there is a considerable amount of data relating to the PCI admission, full procedural details are not recorded on the NIS, which would provide further insight regarding differences in angiographic findings or PCI procedural approaches between men and women. Additionally, no pharmacological information is recorded on NIS, restricting analysis from the use of guideline-directed evidence-based therapies that are known to be underutilized in women and can impact MACE [32]. In keeping with all observational and registry research, the possibility exists of other unmeasured or unrecognized confounders, such as a composite of frailty, which may associate by gender and likely with outcome. Capture of a wide range of comorbid conditions enabling calculation of comorbid burden through the CCI score in the NIS represented a robust objective attempt for comorbidity analysis, although frailty, per se, that is known to associate with poorer outcomes is not captured in this dataset, and is likely to be more prevalent in females who were on average 5 years older than men. Whilst the Charlson score is the most widely used measure of comorbid burden in the literature and has been shown to have an independent prognostic impact on both in-hospital and post discharge outcomes [33], systematic differences in comorbid prevalence between men and women not captured by the Charlson score may bias outcomes. Previous work has suggested that women are less likely to be offered invasive therapy in acute coronary syndromes than men [34] which may lead to imbalances in the risk profile of patients particularly in the ACS setting. Whilst we have attempted to balance for these differences by adjusting for differences in clinical, demographics and comorbid burden, as well as for complexity of PCI (basic fields captured by the NIS such as bifurcation disease, multivessel disease), the NIS dataset does not capture other important measures of risk in this population such as GRACE score. Systematic referral biases may contribute to the adverse outcomes that we report, although it is women at highest risk of ischemic events that are less likely to receive an angiogram / PCI in patients with NSTEMI, meaning that if anything this would tend to favour a better outcome in women. Furthermore, we still report significant sex disparities in PCI cases undertaken for elective indications where such referral biases would be less. Due to the lack of a patient identifier within the dataset, we are unable to identify if the same person appears within the dataset more than once, and so the outcomes reported are based on individual PCI procedures undertaken rather than at the individual patient level. This may introduce bias especially if there are sex differences in the number of repeat procedures and this impacts on outcomes. The NIS dataset does not differentiate between the timing of other diagnoses / equipment to the index PCI event. Therefore in our statistical modelling we have assumed that cardiogenic shock and IABP use were baseline covariates rather than outcome measures following a complication from the procedure which is far less common. Finally, as with any such administrative database, coding errors are always a potential source of bias as is the underreporting of secondary and comorbid diagnosis.

In summary, our study in over 6.5 million PCI discharges across the United States from 2004–2014 indicates that a difference in in-hospital mortality and in-hospital major complications does exist between men and women in real-world practice. These differences persisted despite adjustment for a range of confounders including age and comorbidity. Increased recognition of, and attention to, the likely underlying factors are needed to abolish this disparity.

## Financial disclosure

The authors did not receive any funding for this work.” Danielle Burke is funded by an NIHR School for Primary Care Research Post‐Doctoral Fellowship. The views expressed are those of the author(s) and not necessarily those of the NHS, the NIHR, or the Department of Health”.

## Supporting information

S1 Fig Flow diagram of included records. (TIFF)

S2 Fig Distributions of categorised CCI for men and women for each year of admission for records of patients undergoing PCI. (TIF)

S1 Table Deyo’s modification of Charlson’s co-morbidity index (CCI). (DOCX)

S2 Table ICD-9-CM codes for procedural characteristics. (DOCX)

S3 Table ICD-9-CM codes for post procedural complications. (DOCX)

S4 Table Patient demographics and procedural characteristics for men and women stratified by year of hospitalisation. (DOCX)

S5 Table Odds ratios* and 95% confidence intervals for women versus men in the electively admitted and ACS subgroups. (DOCX)
