# Supplementary figures and images for: Persistent sex disparities in clinical outcomes with percutaneous coronary intervention: Insights from 6.6 million PCI procedures in the United States
Source: PLoS One. 2018 Sep 4;13(9):e0203325. doi: 10.1371/journal.pone.0203325 (PMC6122817; doi:10.1371/journal.pone.0203325)

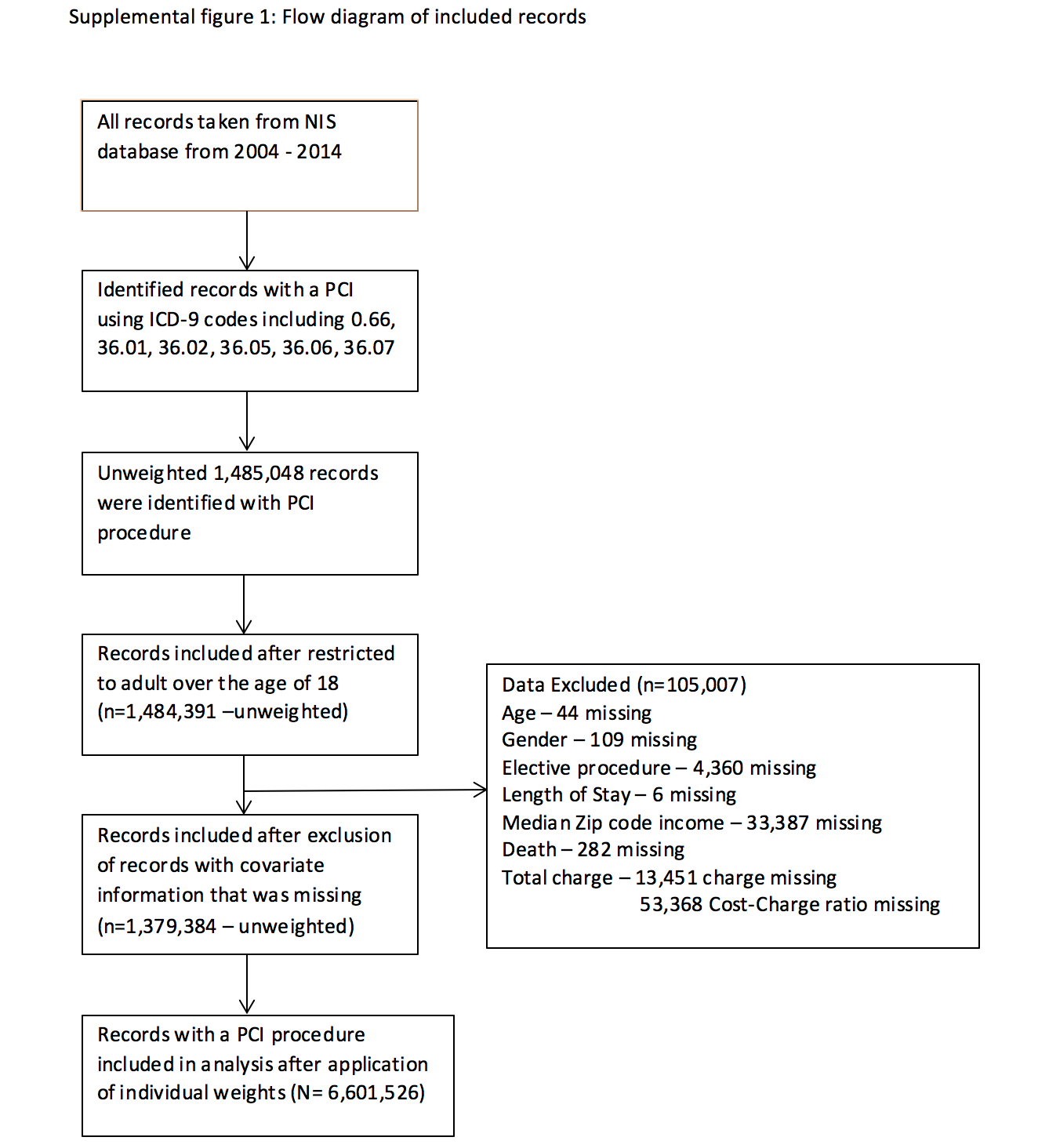

Supplement: S1 Fig — (TIFF) [file pone.0203325.s001.tiff]

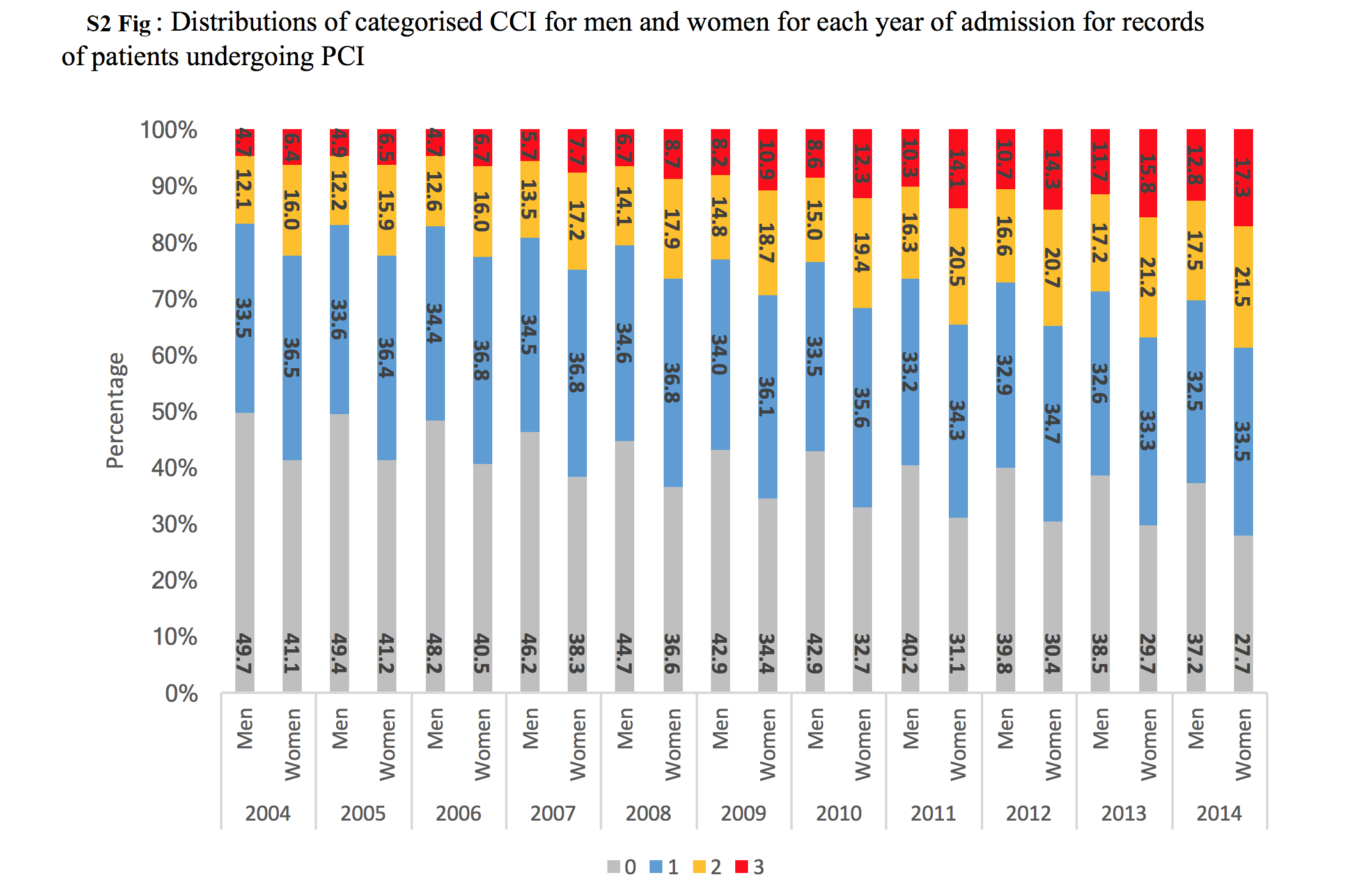

Supplement: S2 Fig — (TIF) [file pone.0203325.s002.tif]
