# Supplementary material for: Persistent sex disparities in clinical outcomes with percutaneous coronary intervention: Insights from 6.6 million PCI procedures in the United States
Source: PLoS One. 2018 Sep 4;13(9):e0203325. doi: 10.1371/journal.pone.0203325 (PMC6122817; doi:10.1371/journal.pone.0203325)
Supplement: S1 Table — (DOCX) [file pone.0203325.s003.docx]

S1 Table: Deyo’s modification of Charlson’s co-morbidity index (CCI).

| Reported ICD-9 codes | Condition | Charlson score |
| --- | --- | --- |
| 412 | Previous myocardial infarction | 1 |
| 428 – 428.9 | Congestive heart failure | 1 |
| 433.9, 441 – 441.9, 785.4 V43.4 | Peripheral vascular disease | 1 |
| V12.54, 438.x | Previous cerebrovascular disease | 1 |
| 290 – 290.9 | Dementia | 1 |
| 490 – 496, 500 –505, 506.4 | Chronic pulmonary disease | 1 |
| 710.0, 710.1, 710.4, 714 – 714.2, 714.81, 725 | Rheumatologic disease | 1 |
| 531 – 534.9 | Peptic ulcer | 1 |
| 571.2, 571.5, 571.6, 571.4 –571.49 | Mild liver disease | 1 |
| 250 – 250.3, 250.7 | Diabetes | 1 |
| 250.4 – 250.6 | Diabetes with chronic complications | 2 |
| 344.1, 342 – 342.9 | Hemiplegia or paraplegia | 2 |
| 582 – 582.9, 583 – 583.7, 585, 586, 588 – 588.9 | Renal Disease | 2 |
| 140 – 172.9, 174 –195.8, 200 – 208.9 | Any malignancy including leukaemia and lymphoma | 2 |
| 572.2 – 572.8 | Moderate or severe liver disease | 3 |
| 196 – 199.1 | Metastatic solid tumour | 6 |
| 042 – 044.9 | AIDS | 6 |
