# Supplementary material for: Persistent sex disparities in clinical outcomes with percutaneous coronary intervention: Insights from 6.6 million PCI procedures in the United States
Source: PLoS One. 2018 Sep 4;13(9):e0203325. doi: 10.1371/journal.pone.0203325 (PMC6122817; doi:10.1371/journal.pone.0203325)
Supplement: S2 Table — (DOCX) [file pone.0203325.s004.docx]

S2 Table: ICD-9-CM codes for procedural characteristics

| **Characteristics** | **ICD-9-CM codes** |
| --- | --- |
| Procedure on a single vessel | 00.40 (36.01 36.02 pre 2005) |
| Procedure on a multiple vessel | 00.41 00.42 00.43 (36.05 pre 2005) |
| Procedure on vessel bifurcation | 00.44 |
| Use of assist device of IABP | 37.6x 97.44 |
| Fractional flow reserve | 00.59 |
| Intravascular ultrasound | 00.24 |
|  |  |
