# Supplementary material for: Persistent sex disparities in clinical outcomes with percutaneous coronary intervention: Insights from 6.6 million PCI procedures in the United States
Source: PLoS One. 2018 Sep 4;13(9):e0203325. doi: 10.1371/journal.pone.0203325 (PMC6122817; doi:10.1371/journal.pone.0203325)
Supplement: S3 Table — (DOCX) [file pone.0203325.s005.docx]

S3 Table: ICD-9-CM codes for post procedural complications

| **Post-procedural Complication** | **ICD-9-CM codes** |
| --- | --- |
| **Bleeding complication** |  |
| Gastrointestinal | 578.9 |
| Unspecified haemorrhage | 459.0 |
| Retroperitoneal haemorrhage | 568.81 |
| Intracranial haemorrhage | 432.9 |
| Intracerebral haemorrhage | 431.x |
| Blood transfusion | V58.2, 99.0x (procedure) |
| **Vascular complications** |  |
| Post-op haemorrhage requiring transfusion | 99.0 (procedure) |
| Vascular injury | 900-904, 998.2, 447, 868.04, 999.7 (diagnosis)  39.31, 39.41, 39.49, 39.52, 39.53, 39.56 - 39.59 39.79 (procedure) |
| **Cardiac complications** |  |
| Iatrogenic cardiac | 997.1 |
| Pericardial comp | 423.0, 423.3 (diagnosis) 47.0 (procedure) |
| Requiring CABG | 36.1x, 36.2, 36.31, 36.32, 36.9x |
| **Post-op stroke/TIA** | 997.00-997.03, 430 – 437.9 |
