# Supplementary material for: Persistent sex disparities in clinical outcomes with percutaneous coronary intervention: Insights from 6.6 million PCI procedures in the United States
Source: PLoS One. 2018 Sep 4;13(9):e0203325. doi: 10.1371/journal.pone.0203325 (PMC6122817; doi:10.1371/journal.pone.0203325)
Supplement: S4 Table — (DOCX) [file pone.0203325.s006.docx]

S4 Table: Patient demographics and procedural characteristics for men and women stratified by year of hospitalisation

| Year | 2004 | | 2005 | | 2006 | | 2007 | | 2008 | |
| --- | --- | --- | --- | --- | --- | --- | --- | --- | --- | --- |
|  | Male | Female | Male | Female | Male | Female | Male | Female | Male | Female |
| PCI discharges | 447,898 | 232,535 | 465,576 | 233,827 | 565,801 | 292,215 | 442,204 | 227,746 | 441,902 | 227,835 |
| Age, median [IQR] | 63 [54,72] | 68 [59,77] | 63 [54,72] | 68 [59,77] | 63 [54,72] | 68 [59,77] | 63 [54,72] | 68 [59,77] | 63 [54,72] | 68 [59,77] |
| Ethnicity |  |  |  |  |  |  |  |  |  |  |
| White | 58.2% | 55.4% | 60.1% | 57.3% | 61.4% | 58.6% | 59.6% | 56.9% | 62.4% | 59% |
| Black | 4.2% | 6.8% | 3.2% | 5.3% | 1.1% | 6.8% | 5.2% | 8.1% | 5.1% | 8.4% |
| Hispanic | 3.6% | 4.2% | 4.8% | 5.2% | 4.7% | 5% | 5.1% | 5.1% | 4.9% | 5.1% |
| Asian/Pacific Islander | 1.2% | 1% | 1.4% | 1.3% | 1.5% | 1.3% | 1.7% | 1.4% | 1.8% | 1.6% |
| Native American | 0.1% | 0.1% | 0.2% | 0.2% | 0.3% | 0.3% | 0.5% | 0.5% | 0.7% | 0.8% |
| Other | 2.1% | 1.8% | 2.7% | 2.3% | 2.5% | 2.1% | 2.7% | 2.3% | 3.5% | 3.1% |
| Missing Information | 30.6% | 30.6% | 27.7% | 28.4% | 25.7% | 25.9% | 25.3% | 25.8% | 21.6% | 22.1% |
| Elective admission | 38.9% | 36.8% | 34.3% | 32.4% | 35.8% | 35.0% | 33.9% | 33.6% | 30.2% | 30.1% |
| Weekday Admission | 87.6% | 87.5% | 87.3% | 87.4% | 87.7% | 88.0% | 86.2% | 86.5% | 85.2% | 85.3% |
| Median ZIP income |  |  |  |  |  |  |  |  |  |  |
| 1st quartile | 22.7% | 28.1% | 24.2% | 29% | 22.2% | 26.3% | 25.2% | 29.7% | 25.3% | 29.5% |
| 2nd quartile | 25.9% | 27.9% | 24.7% | 26.2% | 25.7% | 27.9% | 24.9% | 27.1% | 28.6% | 29.8% |
| 3rd quartile | 24.4% | 23.2% | 26.3% | 25.4% | 26.3% | 25.2% | 24.3% | 23.4% | 24% | 22.7% |
| 4th quartile | 26.9% | 20.8% | 24.8% | 19.4% | 25.9% | 20.6% | 25.5% | 19.8% | 22.2% | 18% |
| Single vessel PCI | 80.8% | 82.5% | 75.3% | 76.5% | 65.8% | 67.1% | 66.7% | 67.2% | 67.1% | 67.3% |
| Multi-vessel PCI | 18.9% | 17.2% | 19.7% | 18.1% | 19.1% | 17.5% | 16.9% | 15.4% | 18% | 17.1% |
| Unknown vessel number | 0.3% | 0.4% | 5.0% | 5.4% | 15.1% | 15.4% | 16.4% | 17.5% | 15.0% | 15.6% |
| Bifurcation stenting | No data | No data | No data | No data | 0.4% | 0.4% | 1.7% | 1.6% | 2.3% | 2.2% |
| Use of assist devise or IABP | 2.7% | 2.4% | 2.5% | 2.4% | 2.5% | 2.3% | 2.9% | 2.7% | 3.3% | 3.1% |
| Shock | 1.5% | 1.8% | 1.6% | 1.9% | 1.7% | 1.9% | 2% | 2.4% | 2.3% | 2.6% |
| Primary diagnosis MI | 32% | 29.6% | 32.1% | 29.7% | 31.6% | 28.8% | 35.8% | 32.6% | 36.6% | 33.7% |
| STEMI diagnosis | 18.8% | 15.7% | 18.5% | 15.6% | 18.4% | 14.9% | 20.7% | 17.1% | 21.1% | 17.3% |
| NSTEMI diagnosis | 15.2% | 16.4% | 15.5% | 16.7% | 15.7% | 17.1% | 18.1% | 19.4% | 19.1% | 20.6% |
| Unstable angina | 25.2% | 27.8% | 24.6% | 26.7% | 22.9% | 24.6% | 21.5% | 24.0% | 22.1% | 23.8% |
| Fractional flow reserve | No data | No data | No data | No data | No data | No data | No data | No data | 0.1% | 0.1% |
| Intravascular ultrasound | 0.6% | 0.6% | 3.4% | 3.7% | 2.9% | 3.3% | 5% | 5.2% | 5.1% | 5.6% |
| Bare Metal Stent | 22.7% | 21.4% | 10.4% | 9.6% | 14.5% | 13.3% | 32.1% | 30.2% | 32.9% | 31.2% |
| Drug Eluting Stent | 74% | 74% | 86.7% | 86.8% | 82.7% | 83% | 64.1% | 65.1% | 62.9% | 63.7% |
| Unknown Stent Type | 6.6% | 7.5% | 5.1% | 5.6% | 5.9% | 6.5% | 7.3% | 7.7% | 7.2% | 7.7% |
| Both stent types used | 3.3% | 2.9% | 2.2% | 2.0% | 3.0% | 2.7% | 3.5% | 2.9% | 3.1% | 2.6% |
| CCI Score |  |  |  |  |  |  |  |  |  |  |
| 0 | 49.5% | 41.1% | 49.4% | 41.4% | 48.2% | 40.5% | 46.2% | 38.3% | 44.5% | 36.41% |
| 1 | 33.6% | 65.5% | 33.5% | 36.3% | 34.4% | 36.8% | 34.5% | 36.8% | 34.6% | 36.6% |
| 2 | 12.2% | 16.0% | 12.1% | 15.9% | 12.6% | 16.0% | 13.5% | 17.2% | 14.1% | 18.1% |
| ≥3 | 4.7% | 6.4% | 4.9% | 6.4% | 4.7% | 6.7% | 5.7% | 7.7% | 6.8% | 8.9% |
| Hypertension | 60.7% | 68.3% | 61.9% | 68.6% | 64.7% | 71.4% | 66.1% | 71.9% | 68.5% | 74.2% |
| Hypercholesterolemia | 19.9% | 18.9% | 16.8% | 16.1% | 14.9% | 14.1% | 14.1% | 14% | 13% | 12.9% |
| Smoking - Yes | 29.3% | 21% | 32.3% | 22% | 33.5% | 24.6% | 35.1% | 26.7% | 37.8% | 28.3% |
| CCI Components |  |  |  |  |  |  |  |  |  |  |
| Previous MI | 35.1% | 33% | 35.1% | 33.3% | 35.3% | 33% | 40% | 37.5% | 41.5% | 39% |
| Heart failure | 10.9% | 16.5% | 10.9% | 16.2% | 11.1% | 16.0% | 12.1% | 16.9% | 12.3% | 16.8% |
| Peripheral vascular disease | 1.2% | 0.9% | 1.1% | 0.9% | 1.3% | 1.0% | 1.4% | 1.1% | 1.5% | 1.2% |
| Previous stroke | 2.0% | 2.9% | 2.1% | 3% | 2.3% | 3.4% | 2.4% | 3.5% | 2.6% | 3.7% |
| Dementia | 0.1% | 0.2% | 0.1% | 0.2% | 0.1% | 0.2% | 0.1% | 0.2% | 0.1% | 0.2% |
| Chronic obstructive disease | 11.7% | 15.9% | 12.4% | 16.6% | 12.7% | 17.3% | 13.4% | 18.7% | 12.9% | 17.8% |
| Connective tissue disease | 0.8% | 2.3% | 0.8% | 3.4% | 0.8% | 2.5% | 0.9% | 2.7% | 0.9% | 2.9% |
| Peptic ulcer | 0.7% | 1.0% | 0.7% | 0.8% | 0.6% | 0.8% | 0.6% | 0.8% | 0.6% | 0.8% |
| Mild liver disease | 0.2% | 0.2% | 0.2% | 0.2% | 0.2% | 0.2% | 0.2% | 0.3% | 0.2% | 0.3% |
| Moderate-severe liver disease | 0.03% | 0.02% | 0.04% | 0.03% | 0.03% | 0.04% | 0.05% | 0.04% | 0.08% | 0.07% |
| Hemiplegia | 0.1% | 0.1% | 0.1% | 0.1% | 0.1% | 0.1% | 0.1% | 0.2% | 0.2% | 0.2% |
| Moderate-severe kidney disease | 0.5% | 0.5% | 0.4% | 0.5% | 0.2% | 0.2% | 0.2% | 0.3% | 0.4% | 0.5% |
| Diabetes – controlled | 24.7% | 30.6% | 25.1% | 30.6% | 25.8% | 31.4% | 27% | 32.5% | 27.9% | 33% |
| Diabetes – uncontrolled | 2.0% | 3.3% | 2.1% | 3.2% | 2.0% | 3.2% | 2.4% | 3.6% | 2.4% | 3.6% |
| Leukaemia & lymphoma | 1.7% | 1.3% | 1.7% | 1.4% | 1.6% | 1.3% | 1.9% | 1.4% | 1.9% | 1.6% |
| Solid tumour + metastasis | 0.2% | 0.3% | 0.2% | 0.3% | 0.2% | 0.3% | 0.3% | 0.3% | 0.3% | 0.3% |
| AIDS | 0.01% | 0% | 0.1% | 0% | 0.1% | 0% | 0.1% | 0% | 0.1% | 0% |

S4 Table continued

| Year | 2009 | | 2010 | | 2011 | | 2012 | | 2013 | | 2014 | |
| --- | --- | --- | --- | --- | --- | --- | --- | --- | --- | --- | --- | --- |
|  | Male | Female | Male | Female | Male | Female | Male | Female | Male | Female | Male | Female |
| PCI discharges | 399,753 | 208,261 | 325,626 | 162081 | 325,281 | 164,684 | 341,264 | 170,455 | 321,385 | 157,540 | 302,400 | 145255 |
| Age, median [IQR] | 63 [54,72] | 68 [59,77] | 62 [54,72] | 68 [58,77] | 63 [54,72] | 68 [58,77] | 63 [55,72] | 68 [58,77] | 63 [55,72] | 67 [58,77] | 63 [55,72] | 67 [58,77]_ |
| Ethnicity |  |  |  |  |  |  |  |  |  |  |  |  |
| White | 66.7% | 64.5% | 66.9% | 64.4% | 68.9% | 66.4% | 73.1% | 70.6% | 73.0% | 70.0% | 73.0% | 71.0% |
| Black | 5.4% | 8.5% | 6.6% | 10.0% | 6.8% | 10.9% | 7.1% | 11.3% | 7.0% | 11.6% | 7.4% | 11.2% |
| Hispanic | 5.4% | 5.5% | 6.3% | 6.0% | 6.3% | 6.3% | 6.6% | 6.5% | 7.0% | 7.1% | 7.2% | 7.0% |
| Asian/Pacific Islander | 1.8% | 1.6% | 2.3% | 2.0% | 2.5% | 1.7% | 2.0% | 1.8% | 2.3% | 1.9% | 2.4% | 1.9% |
| Native American | 0.5% | 1.4% | 0.7% | 0.7% | 0.3% | 0.4% | 0.6% | 0.6% | 0.4% | 1.4% | 0.4% | 0.5% |
| Other | 3.1% | 2.5% | 2.6% | 2.0% | 3.8% | 3.1% | 4.2% | 3.5% | 3.7% | 3% | 3.7% | 2.8% |
| Missing Information | 17.3% | 17% | 14.7% | 14.9% | 11.4% | 11.3% | 6.4% | 6.1% | 6.6% | 6% | 5.9% | 5.6% |
| Elective admission | 24.1% | 24.8% | 21.9% | 21.6% | 20.3% | 20.3% | 18.0% | 18.3% | 15.6% | 15.6% | 11.7% | 11.8% |
| Weekday Day | 83.8% | 84.1% | 81.1% | 81.1% | 80.4% | 80.7% | 80.0% | 80.3% | 78.2% | 79.0% | 76.8% | 77.3% |
| Median ZIP income |  |  |  |  |  |  |  |  |  |  |  |  |
| 1st quartile | 25.4% | 29.8% | 26.1% | 29.9% | 26.4% | 31.3% | 29.0% | 33.9% | 27.5% | 32.5% | 27.4% | 31.4% |
| 2nd quartile | 27.8% | 29.2% | 26.6% | 28.0% | 25.4% | 25.7% | 26.2% | 26.7% | 27.2% | 28.3% | 29.0% | 29.6% |
| 3rd quartile | 25.2% | 23.6% | 25.1% | 24.2% | 26.6% | 25.6% | 23.8% | 22.4% | 24.5% | 22.8% | 23.4% | 22.5% |
| 4th quartile | 21.6% | 17.5% | 22.2% | 17.9% | 21.6% | 17.4% | 20.9% | 17.0% | 20.8% | 16.5% | 20.1% | 16.6% |
| Single vessel PCI | 71.7% | 73% | 68.2% | 69.3% | 74.3% | 74.8% | 76.1% | 76.5% | 76.1% | 77.5% | 75.4% | 76% |
| Multi-vessel PCI | 17.6% | 16.2% | 16.4% | 15.5% | 18.2% | 17.0% | 17.5% | 16.8% | 18.1% | 16.5% | 18.6% | 17.9% |
| Unknown vessel number | 10.7% | 10.8% | 15.4% | 15.2% | 7.6% | 8.2% | 6.4% | 6.7% | 5.5% | 6.0% | 6.0% | 6.1% |
| Bifurcation stenting | 3% | 2.8% | 2.6% | 2.4% | 2.6% | 2.3% | 2.6% | 2.5% | 2.9% | 2.7% | 2.8% | 2.5% |
| Use of assist devise or IABP | 3.7% | 3.0% | 4.1% | 3.6% | 4.3% | 3.6% | 4.4% | 3.8% | 4.4% | 3.7% | 4.7% | 4.3% |
| Shock | 2.8% | 2.9% | 3.4% | 3.7% | 3.7% | 4.0% | 3.8% | 4.1% | 4.2% | 4.5% | 4.9% | 5.5% |
| Primary diagnosis MI | 41.9% | 37.4% | 48.9% | 45.4% | 50.2% | 46.2% | 54.5% | 51% | 58.4% | 55.4% | 63.7% | 61% |
| STEMI diagnosis | 22.6% | 17.6% | 26.3% | 21.7% | 26.3% | 21.2% | 27.2% | 22.4% | 28.3% | 23.4% | 30.3% | 25.2% |
| NSTEMI diagnosis | 22.6% | 24.2% | 25.9% | 28.5% | 27.5% | 29.4% | 30.5% | 32.3% | 33.2% | 36.1% | 36.8% | 40.3% |
| Unstable angina | 21.3% | 23.9% | 20.6% | 22.1% | 20.0% | 22.0% | 19.4% | 20.5% | 18.0% | 19.5% | 16.0% | 17.1% |
| Fractional flow reserve | 0.7% | 0.6% | 0.8% | 0.8% | 1.2% | 1.2% | 2.0% | 2.0% | 2.5% | 2.5% | 2.7% | 3% |
| Intravascular ultrasound | 5.7% | 6.2% | 5.9% | 6.2% | 7.1% | 7.3% | 6.8% | 7.0% | 6.4% | 6.4% | 6.3% | 6.3% |
| Bare Metal Stent | 25.2% | 24.3% | 26.8% | 25.9% | 25.5% | 24.4% | 22.1% | 21.7% | 19.3% | 18.7% | 16.8% | 16.4% |
| Drug Eluting Stent | 69.6% | 70% | 67.6% | 67.6% | 68.6% | 68.9% | 71.9% | 71.5% | 74.3% | 74.3% | 76.5% | 76.3% |
| Unknown Stent Type | 7.2% | 7.4% | 7.3% | 7.8% | 7.6% | 8.0% | 7.2% | 7.9% | 7.5% | 7.9% | 7.8% | 8.2% |
| Both stent types used | 1.9% | 1.7% | 1.7% | 1.3% | 1.6% | 1.3% | 1.1% | 1.1% | 1. % | 0.9% | 1.1% | 0.9% |
| CCI Score |  |  |  |  |  |  |  |  |  |  |  |  |
| 0 | 42.7% | 34.2% | 42.7% | 32.6% | 40.4% | 31.2% | 39.8% | 30.4% | 38.5% | 29.7% | 37.2% | 27.7% |
| 1 | 33.8% | 35.8% | 33.3% | 35.3% | 33.2% | 34.4% | 32.9% | 34.7% | 32.6% | 33.3% | 32.5% | 33.5% |
| 2 | 15.0% | 18.8% | 15.1% | 19.4% | 16.2% | 20.4% | 16.6% | 20.7% | 17.2% | 21.2% | 17.5% | 21.5% |
| ≥3 | 8.5% | 11.1% | 8.9% | 12.6% | 10.2% | 14.0% | 10.7% | 14.3% | 11.7% | 15.8% | 12.8% | 17.3% |
| Hypertension | 69.3% | 74.7% | 70.1% | 75.2% | 71.7% | 76.4% | 72.4% | 77.3% | 72.9% | 77.5% | 73.7% | 78% |
| Hypercholesterolemia | 12.2% | 11.8% | 11.7% | 11.2% | 11.8% | 11.1% | 10.1% | 10.0% | 9.4% | 9.2% | 9.0% | 9% |
| Smoking -Yes | 39.8% | 30.3% | 41.2% | 32.1% | 43% | 33.1% | 45% | 35.8% | 46.3% | 37.5% | 49.2% | 46.5% |
| CCI Components |  |  |  |  |  |  |  |  |  |  |  |  |
| Previous MI | 46.7% | 42.9% | 53.9% | 51.4% | 55.4% | 52.0% | 59.5% | 56.9% | 63.6% | 61.6% | 69.4% | 67.5% |
| Heart failure | 14.0% | 18.5% | 14.9% | 20.7% | 16.7% | 22.3% | 17.4% | 22.5% | 19.1% | 24.5% | 21.0% | 26.3% |
| Peripheral vascular disease | 1.6% | 1.4% | 1.5% | 1.3% | 1.5% | 1.2% | 1.5% | 1.2% | 1.6% | 1.2% | 1.6% | 1.2% |
| Previous stroke | 2.6% | 3.8% | 2.8% | 4.1% | 3.0% | 4.4% | 3% | 4.4% | 3.1% | 4.6% | 3.2% | 4.6% |
| Dementia | 0.2% | 0.2% | 0.1% | 0.2% | 0.1% | 0.3% | 0.1% | 0.2% | 0.1% | 0.2% | 0.1% | 0.3% |
| Chronic obstructive disease | 14.4% | 19.5% | 14.1% | 20.2% | 15.1% | 21.5% | 15.3% | 22.2% | 15.8% | 22.9% | 16.4% | 23.4% |
| Connective tissue disease | 1.0% | 3.1% | 1.1% | 3.3% | 1.1% | 3.6% | 1.2% | 3.6% | 1.2% | 3.8% | 1.3% | 3.9% |
| Peptic ulcer | 0.7% | 1.0% | 0.6% | 0.9% | 0.7% | 0.9% | 0.7% | 0.8% | 0.6% | 0.8% | 0.7% | 0.8% |
| Mild liver disease | 0.3% | 0.3% | 0.3% | 0.3% | 0.4% | 0.3% | 0.4% | 0.3% | 0.4% | 0.5% | 0.5% | 0.5% |
| Moderate-severe liver disease | 0.1% | 0.1% | 0.1% | 0.1% | 0.1% | 0.1% | 0.1% | 0.1% | 0.2% | 0.1% | 0.2% | 0.2% |
| Hemiplegia | 0.2% | 0.2% | 0.3% | 0.3% | 0.3% | 0.3% | 0.2% | 0.4% | 0.3% | 0.4% | 0.4% | 0.4% |
| Moderate-severe kidney disease | 0.5% | 0.8% | 0.7% | 1.1% | 0.8% | 1.1% | 0.8% | 1.0% | 0.9% | 1.3% | 1.0% | 1.5% |
| Diabetes – controlled | 28.1% | 33.7% | 28.3% | 33.9% | 30.0% | 35.5% | 30.3% | 35.8% | 30.4% | 36.1% | 30.6% | 36.4% |
| Diabetes – uncontrolled | 2.8% | 4.3% | 3.1% | 5.0% | 3.7% | 5.5% | 3.9% | 5.6% | 4.1% | 5.8% | 4.5% | 6.8%% |
| Leukaemia & lymphoma | 2.0% | 1.6% | 2.0% | 2.0% | 2.2% | 2.1% | 2.3% | 1.8% | 2.5% | 2.0% | 2.5% | 2.3% |
| Solid tumour + metastasis | 0.3% | 0.3% | 0.4% | 0.4% | 0.4% | 0.4% | 0.4% | 0.4% | 0.4% | 0.4% | 0.4% | 0.4% |
| AIDS | 0.2% | 0% | 0.2% | 0.1% | 0.1% | 0.1% | 0.1% | 0% | 0.2% | 0% | 0.2% | 0.1% |
