# Supplementary material for: Persistent sex disparities in clinical outcomes with percutaneous coronary intervention: Insights from 6.6 million PCI procedures in the United States
Source: PLoS One. 2018 Sep 4;13(9):e0203325. doi: 10.1371/journal.pone.0203325 (PMC6122817; doi:10.1371/journal.pone.0203325)
Supplement: S5 Table — (DOCX) [file pone.0203325.s007.docx]

S5 Table: Odds ratios* and 95% confidence intervals for women versus men in the electively and ACS subgroups.

|  | Elective population  (n=1,789,744) | ACS diagnosis  (n=4,434,750) |
| --- | --- | --- |
|  | Odds Ratio (95% CI) | Odds Ratio (95% CI) |
| In-hospital mortality | 1.40 (1.25,1.57) | 1.18 (1.14,1.22) |
| Any complication | 1.34 (1.30, 1.38) | 1.35 (1.33,1.38) |
| Bleeding complication | 1.95 (1.85,2.06) | 1.77 (1.73,1.82) |
| Vascular complication | 1.57 (1.47,1.68) | 1.54 (1.48,1.61) |
| Cardiac complication | 1.04 (0.99, 1.09) | 0.96 (0.94,0.99) |
| Post-operative stroke | 1.17 (1.13,1.23) | 1.27 (1.23,1.31) |

*Adjustment for age, median income, elective admission, day of admission (weekend/weekday), primary diagnosis of MI, diagnosis of STEMI/ NSTEMI or unstable angina, diagnosis of shock, hypertension, or hypercholesterolemia, patient smoking status, Charlson comorbidities, previous PCI, previous CABG, use of an assist device or IABP, use of a bare metal or drug eluting stent, bifurcation stenting, fractional flow reserve, single or multi-vessel PCI and year of hospitalisation
